# *In silico* study for diversing the molecular pathway of pigment formation: an alternative to manual coloring in cotton fibers

**DOI:** 10.3389/fpls.2015.00751

**Published:** 2015-09-17

**Authors:** Ammara Ahad, Aftab Ahmad, Salah ud Din, Abdul Q. Rao, Ahmad A. Shahid, Tayyab Husnain

**Affiliations:** Center of Excellence in Molecular Biology, University of the PunjabLahore, Pakistan

**Keywords:** anthocyanin, DFR, delphinidin, cytochromes and flavonoid 3′, 5′-hydroxylase

## Abstract

Diversity of colors in flowers and fruits is largely due to anthocyanin pigments. The flavonoid/anthocyanin pathway has been most extensively studied. Dihydroflavonol 4-reductase (DFR) is a vital enzyme of the flavonoid pathway which displays major impact on the formation of anthocyanins, flavan 3-ols and flavonols. The substrate specificity of the DFR was found to play a crucial role in determination of type of anthocyanidins. Altering the flavonoid/anthocyanin pathway through genetic engineering to develop color of our own choice is an exciting subject of future research. In the present study, comparison among four DFR genes (*Gossypium hirsutum, Iris* × *hollandica, Ang. DFRI* and *DFRII*), sequence alignment for homology as well as protein modeling and docking is demonstrated. Estimation of catalytic sites, prediction of substrate preference and protein docking were the key features of this article. For specific substrate uptake, a proline rich region and positions 12 plus 26 along with other positions emphasizing the 26-amino acid residue region (132–157) was tested. Results showed that proline rich region position 12, 26, and 132–157 plays an important role in selective attachment of DFRs with respective substrates. Further, “Expasy ProtParam tool” results showed that *Iris* × *hollandica* DFR amino acids (Asn 9: Asp 23) are favorable for reducing DHQ and DHM thus accumulating delphinidin, while *Gossypium hirsutum* DFR has (Asn 13: Asp 21) hypothesized to consume DHK. Protein docking data showed that amino acid residues in above mentioned positions were just involved in attachment of DFR with substrate and had no role in specific substrate uptake. Advanced bioinformatics analysis has revealed that all above mentioned positions have role in substrate attachment. For substrate specificity, other residues region is involved. It will help in color manipulations in different plant species.

## Introduction

More than 200,000 different types of compound have been shown to be produced collectively by higher plants, and some of these are able to generate bright colors in flowers, fruit or foliage (Feng et al., 2013). The human eye can detect light, as reflected or transmitted by a compound under wavelengths 380 and 730 nm, while insects recognize the light of shorter wavelengths (Davies, 2004). In plants, three major classes of pigments for coloration exist which includes carotenoids, flavonoids/anthocyanins, and betalains.

Diversity of red, purple and blue colors of flowers as well as fruits are due to the nature of these polyphenolic pigments. In plants, these secondary metabolites are found ubiquitously. Among factors influencing flower color, the flavonoid/anthocyanin biosynthesis has been most extensively studied. The various factors which influence the final color formation includes anthocyanin structures, vacuolar pH, co-pigments and metal ions.

In higher plant species, the pathway leading to anthocyanidin 3-glucoside is generally conserved (Grotewold, 2006; Tanaka and Brugliera, 2006b). Only six major classes of anthocyanidins do exist i.e., pelargonidin, cyanidin, peonidin, delphinidin, petunidin, and malvidin (Yoshida et al., 2009) Among cytochromes P450s, two genes, i.e., flavonoid 3′-hydroxylase (F3′H) and flavonoid 3′, 5′-hydroxylase (F3′5′H), catalyze hydroxylation of the B-ring (Tanaka, 2006a). The increased hydroxylation pattern of this ring was found to be involved in shifting the anthocyanin color toward blue. They exhibit broad substrate specificity and catalyze hydroxylation of flavanones, dihydroflavonols, flavonols, and flavones. Flavanones along with dihydroflavonols are precursors of anthocyanidins and anthocyanins. Trihydroxylated delphinidin based anthocyanins from blue or violet colors is achieved by presence of F3′5′H (Honda and Saito, 2002).

Dihydroflavonol 4-reductase (DFR) is a vital enzyme of the flavonoid pathway which shows a major impact on the formation of anthocyanins, flavan 3-ols, and flavonols. In ornamental flower plants, the color has been modified by altering the expression levels of DFR genes (Aida et al., 2000). The substrate specificity of the DFR plays a crucial role in determining which anthocyanidins, a plant will accumulate (Forkmann and Heller, 1999).

DFR is unique in a sense that it uptakes flavoniod substrates depending on the B-ring hydroxylation pattern. The DFRs of several plants accept dihydroflavonols having one (dihydrokaempferol, DHK), two (dihydroquercetin, DHQ), or three (dihydromyricetin, DHM) hydroxyl groups on the B-ring. NADPH is used as a cofactor of DFR, which helped in catalyzing the reduction of dihydroflavonols to leucoanthocyanidins which were common precursors of anthocyanin (Helariutta et al., 1993; Tanaka et al., 1995; Dellus et al., 1997). Substrate specificity of DFR determines the conversion of metabolic flux toward desired anthocyanidin biosynthesis. For example in Petunia hybrid, the transformation of a DFR from maize evaded the gap in the pathway of anthocyanin formation and successfully generated orange color flower by the reduction of DHK and the accumulation of pelargonidin anthocyanins. Similarly, petunia or viola F3′5′H gene in combination with the petunia DFR gene was transformed in cultivars of white carnation that specifically missing DFR gene, hence resulted in violet carnations by the generation of delphinidin based anthocyanins (Holton, 1996; Fukui et al., 2003).

The two most common fiber colors in colored cotton cultivars (*Gossypium hirsutum*) are “brown and green.” They are eco-friendly, economical, and valuable for human health (Zhu et al., 2006) Four key genes of the flavonoid biosynthetic pathway (*GhC4H, GhCHS, GhF3*′*H*, and *GhF3*′*5*′*H, GhDFR*) have been reported in *Gossypium hirsutum*.

The idea of pigment engineering, for diversity of colors in the cotton fiber, can be opened up by investigating the mechanism of pigmentation. The key is to explore and understand the molecular basis of pigment formation and its deposition in fibers (Feng et al., 2013). The purpose of this research was to investigate the putative involvement of flavonoid biosynthesis pathway structural gene (DFR) in the pigmentation and coloration of cotton fiber.

To achieve this objective a comparison was made in retrieving sequences of two DFR genes from blue flower species and two from white including sequence alignment for homology and protein modeling. Further more the presence of proline rich region along with positions 12 and 26 was reported to be important in determining substrate specificity for DFR of *A. angustifolia* (Gosch et al., 2014). Other positions emphasizing the 26-amino acid region (132–157) were also mentioned in the literature which played a role in utilizing specific dihydroflovonols: DHK, DHQ, and DHM (Johnson et al., 2001; Xie et al., 2004). It was tested in *Gossypium hirsutum* as well as in *Iris* × *hollandica* by protein-ligand docking analysis. Analysis of gene cluster encoding dihydroflavonol 4-reductases in the *Lotus japonicus* genome showed that three out of six DFR proteins exhibit catalytic activity, their substrate preferences settled with the variation of a specific active site residue (Aspartic acid or Asparagine) and found to be involved in controlling the substrate specificity (Shimada et al., 2005).

The structure of the DFR proteins (*Gossypium hirsutum* as well as in *Iris* × *hollandica*) have not yet determined experimentally (NMR or X-ray), and for that reason models were built by following homology modeling and threading protocol. Moreover, for the estimation of respective catalytic sites and prediction of substrate preference, protein docking was performed. The aim of this study was to identify the region that involves in the substrate specificity of DFR to give readers an idea of manipulating the pathways involved in color modification, so that *Gossypium hirsutum* fiber pigmentation may be altered, similarly as reported in the case of some ornamental flower plants.

## Materials and methods

*In silico* analysis was carried out in order to evaluate substrate specificity of different types of DFRs (dihydromyricetin reductase, dihydroquercetin reductase and dihydrokaempferol 4-reductase). For this assessment, dihydroflavonol 4-reductase (DFR) sequence information available in NCBI database was used. Protein sequence alignment, designing of protein structures, ligand structure retrieval and molecular docking was also carried out.

### Determination of substrate binding region among different plant species

All above mentioned positions (12, 26 and from 132 to 157) were evaluated for the presence of particular residues and its role in specific substrate uptake as illustrated in published data. For this purpose full length four DFRs sequences: *Angelonia angustifolia, Ang. DFRI* (GenBank. AIR09398.1), *Ang. DFRII* (GenBank. AHM27144.1), *Gossypium hirsutum* (GenBank. AHG97389.1), and *Iris* × *hollandica* (GenBank. BAF93856.1) were retrieved from Genbank. Sequences were arranged by using “Bioedit” program to find out the positional similarities between the residues of these sequences. To further validate the role of this particular position in substrate specificity, sequences from five other species were taken which includes *Rosa chinensis* (GenBank. AHF58604.1), *Vaccinium macrocarpon* (GenBank. AF483835.1), *Gerbera hybrid* (GenBank.CAA78930.1), *Petunia* × *hybrid* (GenBank. AF233639.1), and *Ampelopsis grossedentata* (GenBank. AGO02174.1). These nine sequences were aligned by using the CLC Genomics Workbench 8.

To evaluate substrate specificity, Asn as well as Asp percentage estimation in DFR sequences for *Iris* × *hollandica* and *Gossypium hirsutum* was done by using “Expasy ProtParam tool.”

### Modeling of receptor molecules for docking analysis

Protein sequences of *Gossypium hirsutum* and *Iris* × *hollandica* (retrieved from NCBI) were used for 3D modeling as their protein structures were not available on protein structure databases. For modeling purposes, sequences were submitted to I-TASSER server (http://zhanglab.ccmb.med.umich.edu/I-TASSER/). This tool produced a protein model based on homology modeling and threading. For homology modeling of *Gossypium hirsutum* DFR, the PDB templates used were PDB: 2C29F (Identity 82%, coverage 92%) and PDB: 2C29A(Identity 82%, coverage 91%). Whereas, for protein modeling of DFR (*Iris* × *hollandica*) PDB: 2C29F (Identity 66%, coverage 90%) and PDB: 2C29A (Identity 67%, Coverage 90%) template were used.

### Refinement and evaluation of DFR protein model

The model was further refined by using online tool, ModRefiner accessed on Zhang Lab website (http://zhanglab.ccmb.med.umich.edu/ModRefiner/). This tool minimized the energy of the model and took the residues of protein in the allowed region. The models were evaluated and validated by producing Ramachandran plot. These plots were plotted by using the online RAMPAGE tool (http://mordred.bioc.cam.ac.uk/~rapper/rampage.php). Ramachandran plots of proteins determined their stability.

### Ligand preparation

The structures of biological compounds of flavonoid pathway dihydrokaempferol (PubChem: 122850), dihydroquercetin (PubChem: 439533), and dihydromyricetin (PubChem: 161557) were downloaded from the PubChem database in 2D format. For preparation of ligand structures which were used in docking, hydrogen atoms were added to each ligand and their energy was minimized by the means of MMFF94X force field at 0.05 gradients. Then, these ligand structures were saved in.mol2 file format. A database of ligands (DFR acceptor compounds) was created in MOE software.

### Protein-ligand docking of DFR protein and ligand molecules

Three-dimensional model of *Gossypium hirsutum* and *Iris* × *hollandica* were constructed by using I-TASSER server. The water molecules were removed with the help of MOE software. After the removal of water molecules, hydrogen atoms were added to the receptor proteins. Optimization of receptor molecule was achieved by energy minimization and 3D protonation (with help of AMBER99 force field option of MOE). The gradient was 0.05 and receptor was minimized unless root mean square gradient fall below 0.05. After 3D protonation of the receptor protein, the hydrogen molecules were hidden. This energy minimized, 3D protonated receptor molecules were then used for docking analysis. Box of 26 residues as reported by Shimada et al. (2005) was aligned with *Iris* × *hollandica* and *Gossypium hirsutum*. These aligned residues were used as pocket site.

Molecular docking was carried out against the databases mentioned previously, after the modeling and preparation of ligand and receptor molecules. The receptor residues in region 132–157 of *Gerbera* DFR correspond to 148–174 residues in *G. hirsutum* as well as 127–153 in *Iris* × *hollandica* were selected and docked with ligands. Docking output database file having receptor ligand complex was saved in.mdb format. The docked complexes were categorized with increasing *S*-value (Final score to indicate binding free energy). The complexes with minimum S were taken to evaluate the interactions of ligand with the active site residues of the receptor proteins. Best hydrogen bonding plus π-π interactions were evaluated by the using ligX option of MOE.

## Results

### Comparison of DFR reported residues involved in substrate specificity

**Figure 1:**
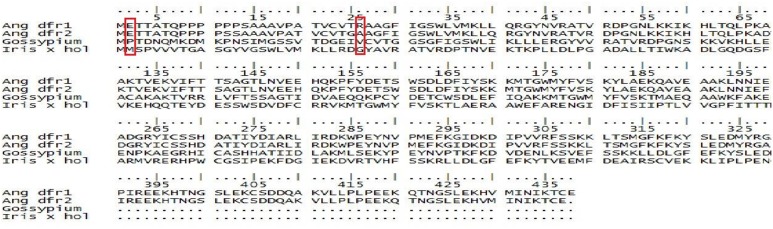
**Alignment of the amino acid sequences encoded by *Ang.DFRI and DFRI*I, *Gossypium hirsutum*, *and Iris* × *hollandica***. Proline rich region is marked with a box.

**Figure 2:**
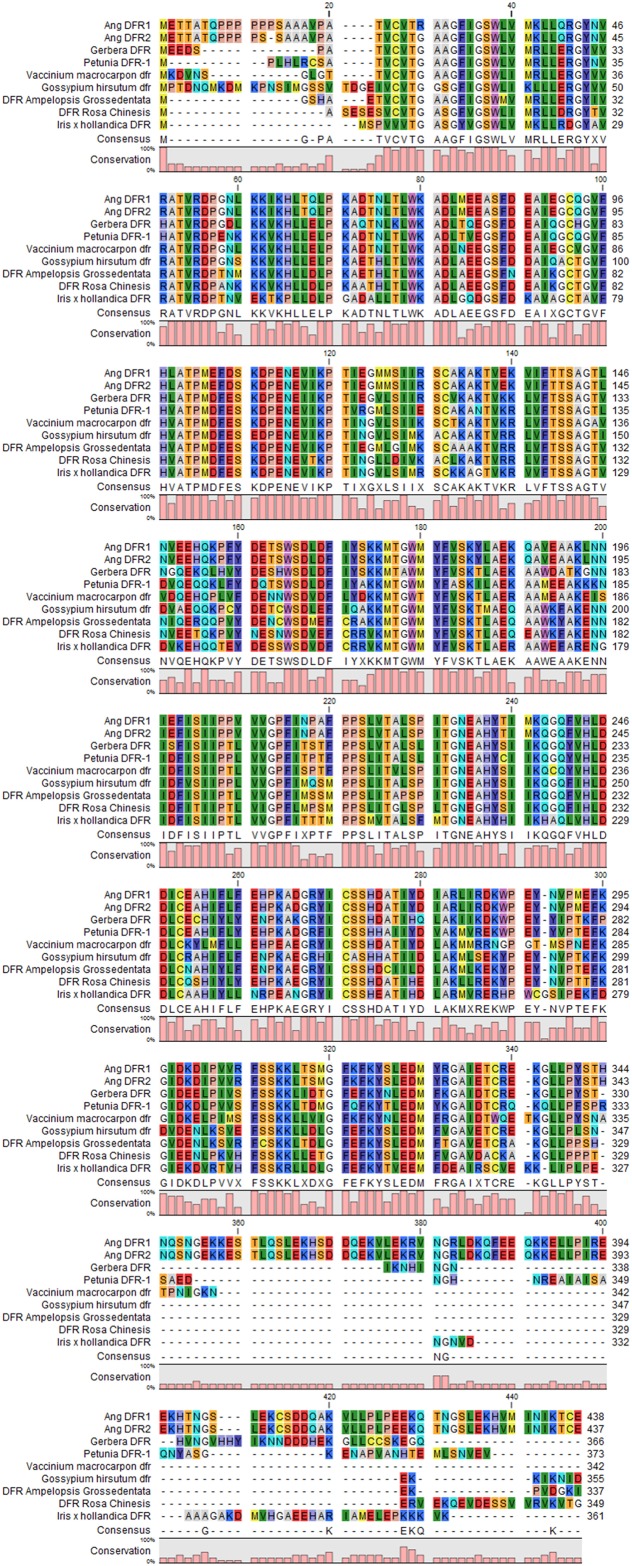
**Multiple sequence alignment of dihydroflavanol 4-reductase, consensus sequences of different plant species (*Rosa chinensis*, *Vaccinium macrocarpon*, *Gerbera hybrid, Petunia* × *hybrid, Ang. DFRI, Ang. DFRII, Gossypium hirsutum, Iris* × *hollandica, and Ampelopsis grossedentata*) were achieved by using CLC Genomics Workbench 8**. The colored bars at the bottom are representing the conservation Percentage.

For comparison, at position 12 and 26, DFR sequences were aligned by using the CLC Genomics Workbench 8 as mentioned earlier. From sequence alignment results it was evaluated that at position 12, *Ang. DFRI, Ang. DFRII, Gossypium hirsutum* DFR, and *Iris* × *hollandica* DFR have proline, serine, proline and glycine respectively. This result showed same residue (proline) in both *Gossypium hirsutum* and *Angelonia DFR I* whereas, *Angelonia DFRII* and *Iris* × *hollandica* DFR had serine and glycine which were functionally similar residues (Figure 1). In present study, the sequence alignment showed proline at 12 position in *Gossypium hirsutum* from which it is hypothesized that it would reduce dihydrokaempferol. Whereas, this particular region is absent in both *Iris* × *hollandica* and *Ang.II* DFRs, resulting in delphinidin accumulation which is responsible for production of blue color (Figure 2). For further confirmation DFRs from five more species along with these four plant species were carried out to evaluate results. However, with respect to *Ang. DFRI*, sequence alignment of other five species (*Rosa chinensis, Vaccinium macrocarpon, Gerbera hybrid, Petunia* × *hybrida*, and *Ampelopsis grossedentata*) showed the deletion of proline rich region and thus unable to reduce DHK.

On 26th position regarding *Ang.DFRI* has arginine and all other species DFR have glycine (position 25 in *Ang.DFRII*,13 *Rosa chinensis*, 16 *Vaccinium macrocarpon*, 13 *Gerbera hybrid*, 15 *Petunia*×*hybrida*, 12 *Ampelopsis grossedentata*, and 30 in *Gossypium hirsutum* DFR (Figure 2). In DFRs, where proline is absent at position 12, it would show glycine on that particular or nearby position. Thus, it is speculated that when a proline rich region is absent, the DFRs would reduce DHQ or DHM as substrate. Results showed that proline rich region in general and position 12 in particular plays main role in selective attachment of these reductases with their relative specific substrates (DHK).

### Role of Asn and Asp type DFRs in substrate specificity

Recent analysis on DFRs (*Iris* × *hollandica* and *Gossypium hirsutum*) was done by “Expasy ProtParam tool” in order to get information about the presence of total Asn and Asp residues in them (Table 1A). Results showed that *Iris* × *hollandica* DFR has (Asn 9: Asp 23) while *Gossypium hirsutum* DFR has (Asn 13:Asp 21) (Table 1B). This is postulated that *Iris* × *hollandica* DFR (Asp-type) has more likeness to reduce DHM in comparison to DHK hence it has a key role in accumulating delphidin by utilizing DHM as substrate.

**Table 1:** **Amino acid percentage in both *Gossypium hirsutum and Iris* × *hollandica* (Asn,9:Asp,23) by using Protparam tool**.

**A**			**B**		
Number of amino acids: 361			Number of amino acids: 355		
Molecular weight: 40221.2			Molecular weight: 39651.7		
Theoretical pI: 6.13			Theoretical pI: 5.67		
Amino acid composition:			Amino acid composition:		
Ala (A)	32	8.9%	Ala (A)	25	7.0%
Arg (R)	18	5.0%	Arg (R)	8	2.3%
Asn (N)	9	2.5%	Asn (N)	13	3.7%
Asp (D)	23	6.4%	Asp (D)	21	5.9%
Cys (C)	7	1.9%	Cys (C)	8	2.3%
Gln (Q)	4	1.1%	Gln (Q)	9	2.5%
Glu (E)	26	7.2%	Glu (E)	27	7.6%
Gly (G)	22	6.1%	Gly (G)	20	5.6%
His (H)	13	3.6%	His (H)	9	2.5%
Ile (I)	19	5.3%	Ile (I)	25	7.0%
Leu (L)	25	6.9%	Leu (L)	29	8.2%
Lys (K)	25	6.9%	Lys (K)	32	9.0%
Met (M)	14	3.9%	Met (M)	13	3.7%
Phe (F)	15	4.2%	Phe (F)	18	5.1%
Pro (P)	17	4.7%	Pro (P)	18	5.1%
Ser (S)	22	6.1%	Ser (S)	25	7.0%
Thr (T)	23	6.4%	Thr (T)	20	5.6%
Trp (W)	6	1.7%	Trp (W)	5	1.4%
Tyr (Y)	8	2.2%	Tyr (V)	7	2.0%
Val (V)	33	9.1%			
Pyl (O)	0	0.0%			
Sec (U)	0	0.0%			

### Modeling, refinement, evaluation and validation of DFR protein

**Figure 3:**
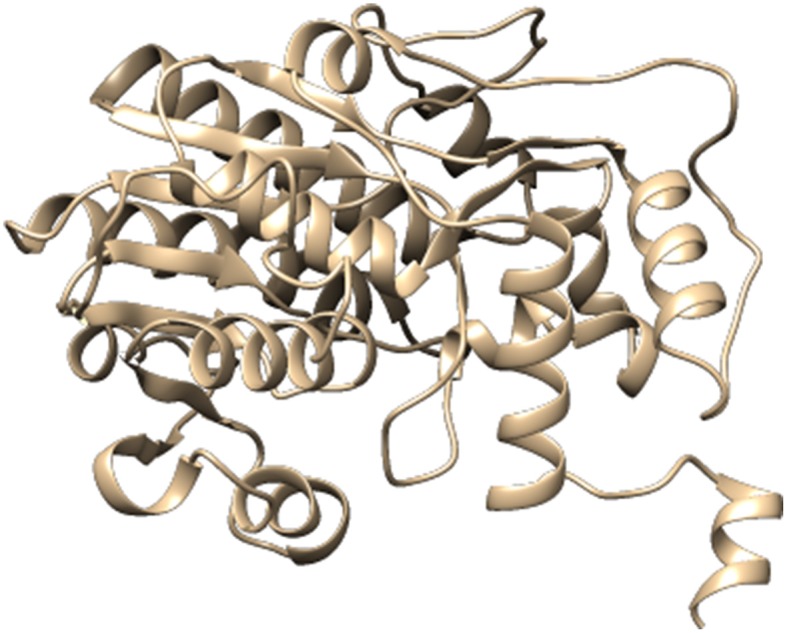
**Three dimensional protein model of *Gossypium hirsutum* DFR protein, predicted by I-TASSER**.

**Figure 4:**
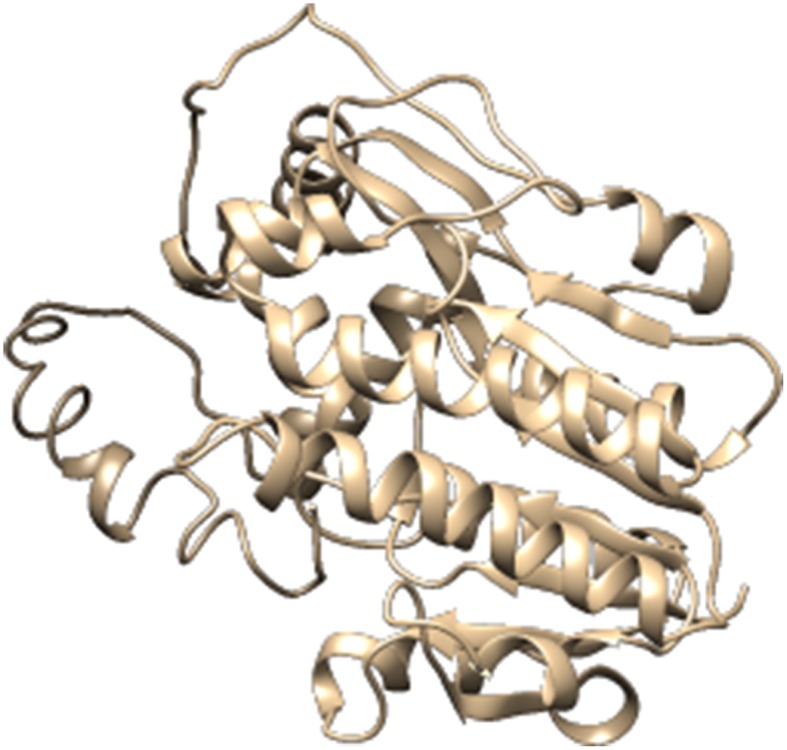
**Three dimensional protein model of and *Iris* × *hollandica* protein, predicted by I-TASSER**.

For the construction of 3D Model of DFR protein, retrieved sequences of both *Iris* × *hollandica* DFR and *Gossypium hirsutum* DFR were submitted to online I-TASSER server (Figures 3, 4).

**Figure 5:**
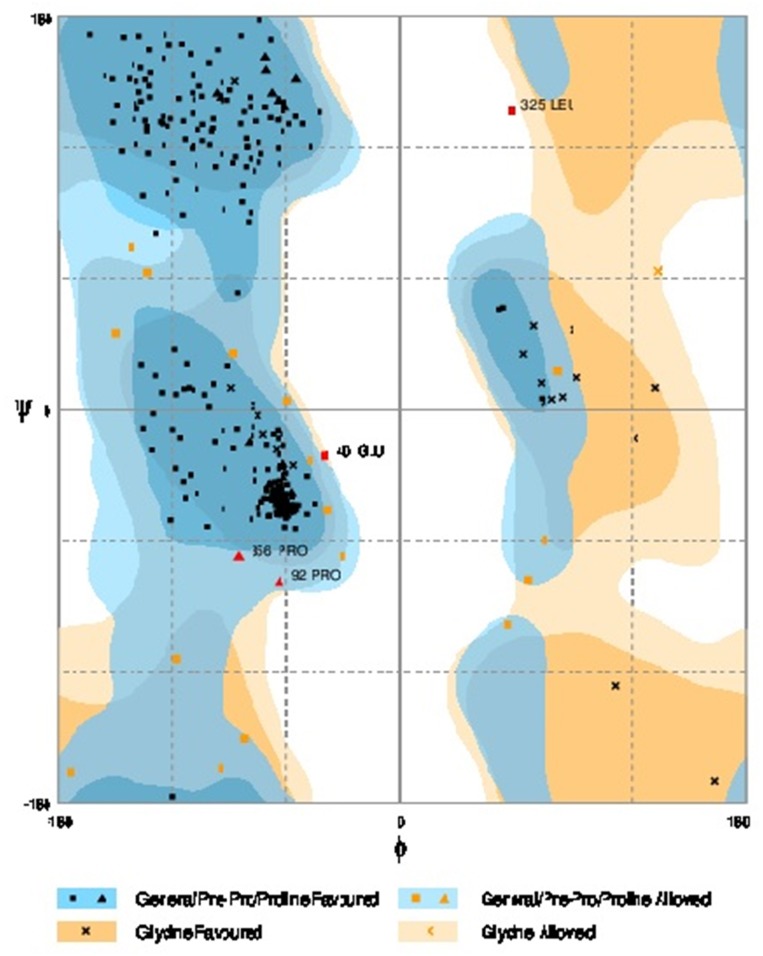
**Ramachandran plot analysis of *Iris* × *hollandica* model to visualize dihedral angles; φ against ψ**. At the bottom of the image the summary of evaluating residues is presented.

**Figure 6:**
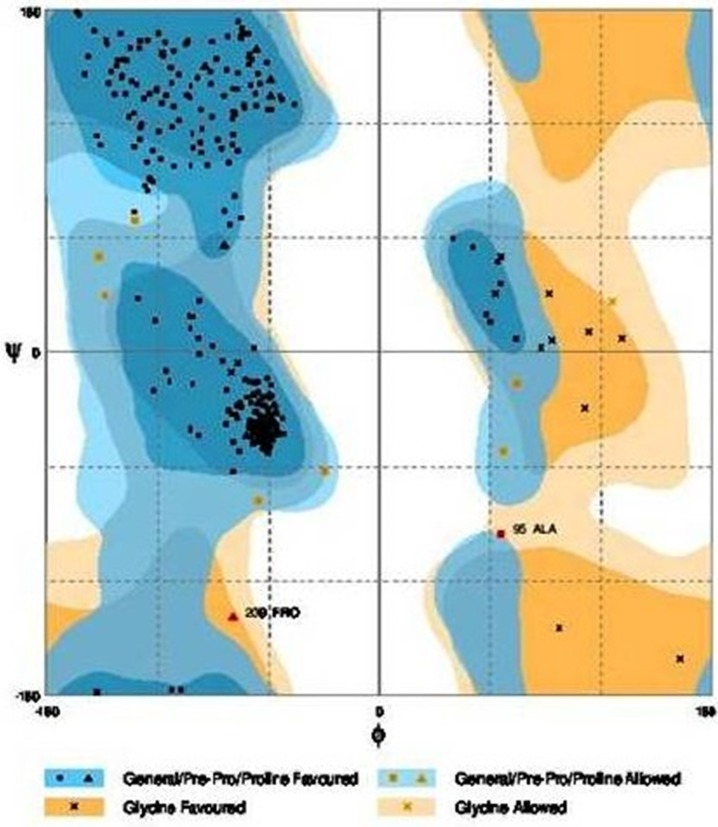
**Ramachandran plot analysis of *Gossypium hirsutum* protein model to visualize dihedral angles; φ against ψ**. At the bottom of the image the summary of evaluating residues is presented.

Further refinement was achieved by using the ModRefiner tool. The constructed models were subjected to RAMPAGE to create Ramachandran Plot for model evaluation. Figures 5, 6 showed a Ramachandran plot of the Dihydroflavonol 4-Reductase protein's model. Plot displayed the presence of 343 (97.2%) residues in favored region, 8 (2.3%) residues in allowed region while 2 (0.6%) residues in outlier region in case of *Gossypium hirsutum* DFR (Figure 6) and 338 (94.2%) residues in favored region 17 (4.7%) residues in allowed region plus 4 (1.1%) residues in outlier region in Iris DFR (Figure 5). These computed results validated the models because for a fine model more than 90% residues should be in both favored and allowed region.

### Protein-ligand docking results

**Figure 7:**
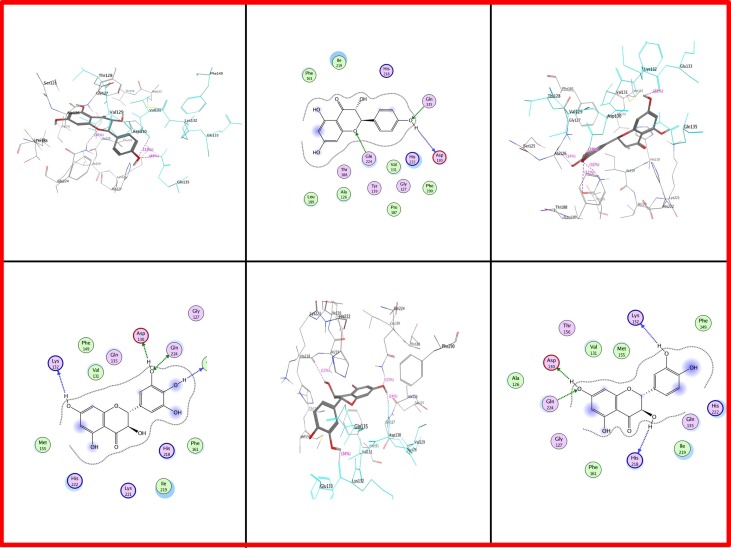
**Two and three dimensional interaction diagrams of DFR *Iris* × *hollandica* with dihydroflavonols**. Interaction diagrams were attained by using ligand interaction analysis feature of MOE.

Docking results in case of *Iris* × *hollandica* showed that position 130 has Asp as well as Gln in all: DHK, DHQ, and DHM. Additional Glutamine at position 135 also showed attachment with DHK. However, Lys at positions 132 (of *Iris* × *hollandica* DFR) has been engaged in DHM and DHQ binding. Ala in position 126 (DHM) and His 218 (DHQ) had an impact on substrate binding (Figure 7).

**Figure 8:**
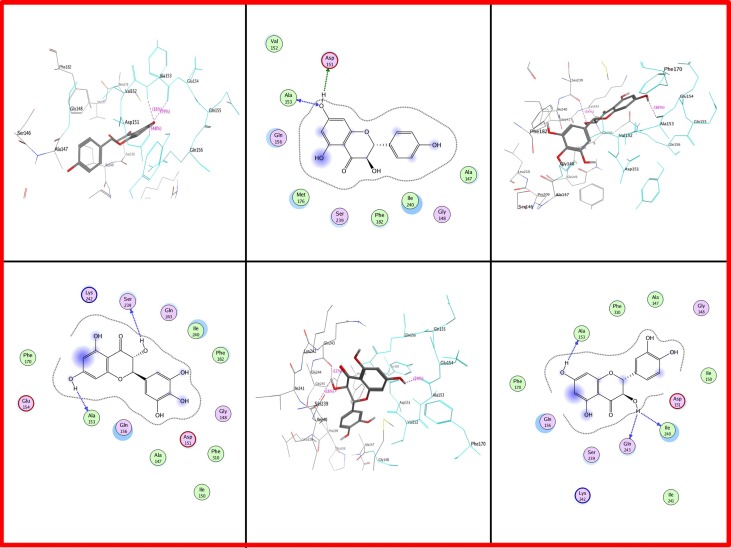
**Two and three dimensional interaction diagrams of *Gossypium hirsutum* with dihydroflavonols**. Interaction diagrams were attained by using ligand interaction analysis feature of MOE.

As far as *Gossypium hirsutum* DFR is concerned, Ala at position 153 is present in all types of dihydroflavonols. Asp at position 151 is involved in *Gossypium hirsutum* DFR binding with DHK (Figure 8). Serine is positioned at 239 which showed interaction in DHM while lle at 240 is involved in DHQ.

From all data gathered from these proteins docking it was concluded that all above mentioned residues at defined particular positions were just involved in attachment with dihydroflavonols (DHK, DHQ, and DHM) and they have no role in the specificity of substrates. Present work showed that 26 residue region was highly variable in DFRs from different plant species.

## Discussion

The substrate specificity of DFR has been widely studied in a range of plants (Hua et al., 2013) Dihydroflavonol 4-reductases from seven different species (*Ang.DFRII, Rosa chinensis, Vaccinium macrocarpon, Gerbera hybrid, Petunia*×*hybrida, Ampelopsis grossedentata*, and *Iris* × *hollandica*) displayed deletion of the proline rich region and had glycine mostly in close proximity or at position 12 in comparison of position 26 of *Ang. DFRI*. Therefore, most of these DFRs could be able to reduce DHQ or DHM but not DHK. Results matched with pervious studies done by Johnson et al. (2001) who reported inability of petunia DFR to reduce DHK but with the transformation of maize DFR in petunia generated orange colored flowers showing DHK reduction (Meyer et al., 1987). Although between *Ang*. *DFRI* and *DFRII* sequences, 99% identity exist, yet position 12 and 26 was reported to be crucial in substrate specificity. Findings were also similar to Gosch and his co-workers who documented proline rich region near N-terminus and same residue at position 12 while glycine at position 26 (in all DFRs except *Ang.DFRI*) andfurther emphasized on alteration of *AngI/II DFR* functional activity by interfering with this region (Gosch et al., 2014). However, the region was a proline rich motif that acts as a NADPH binding site (Petit et al., 2007). Presence of proline at 12 position in *Gossypium hirsutum* DFR showed ability to reduce dihydrokaempferol. Recent results indicate that the reduction of dihydrofavonols by DFR can be a significant enzymatic step for flower color determination, by DHK hydroxylation medicated by F3′H or F3′5′H. Those DFRs which have the ability to consume DHQ along with DHM as substrates could produce red or blue flowers. Absence of proline rich region in DFRs would reduce DHQ and DHM as substrate. Results were in harmony with Gosch et al. (2014) who reported blue color generation by the replacement of arginine residue by glycine in *Ang.DFRI* and mutation of proline at 12 position. He further explained that variability among amino acid sequences in the particular region or variation in substrate specificity by a single mutation in a particular area in DFRs from different plant species or even in different cultivars of the same species could have different substrate preferences. In another report, aspartic acid at position 134 in petunia showed its ability to utilize DHM thus had blue colored flowers (Johnson et al., 2001). These contradictions in results may be due to species and amino acid residue differences. All mentioned Docked protein showed that respective residues were involved in substrate attachment and not in specificity of particular substrate. Moreover, the complete loss of *Ang.DFRII* activity with no production of blue color flowers was observed by site mutagenesis created by the replacement of glycine with arginine at position 12 (Gosch et al., 2014). Recent work showed increased aspartic acid residues in *Iris* × *hollandica* DFR which favors DHM as substrate in comparison to *Gossypium hirsutum* DFR having asparagines residues hence unable to reduce DHM and able to reduce DHK. Current findings were in support with Shimada et al. (2005) who reported that out of six DFR proteins, the DFR2 and DFR3 (Asn-type DFRs) showed increased uptaking of DHK than DHQ as substrate whereas DFR5 (Asp-type) reduces very less DHK in the Lotus japonicus. Previous research enlightened that amino acid residue differences caused different substrate preferences therefore, transformation of Iris DFR in rose reduced DHM as substrate and generated blue hued flowers whereas *Gossypium hirsutum* DFR reduced DHK had brown shade color lint (Katsumoto et al., 2007). Similar findings had been documented in *M. Truncatula* where DFR (Asp-type) reduces DHK minutely (Xie et al., 2004). It is anticipated that by the transformation of *Iris* × *hollandica* DFR in *Gossypium hirsutum* blue pigmentation could be achieve in fiber. Moreover, from all these *in silico* analysis it is hypothesized that by the transformation of specific DFR which reduces its relative substrate different colored phenolic pigments can be deposited in cotton fiber and hence colored cotton of desired colors will be generated in future. This approach presents first report to generate ecofriendly colored cotton.

## Author contributions

Ammara A participated in the experimental design; material collection including manuscript right up, Aftab A participated in the bioinformatics analysis, SD helped in writing the manuscript. All other authors read and approved the final manuscript.

### Conflict of interest statement

The authors declare that the research was conducted in the absence of any commercial or financial relationships that could be construed as a potential conflict of interest.

## Abbreviations

DHM: dihydromyricetin reductase
DHQ: dihydroquercetin reductase
DHK: dihydrokaempferol 4-reductase
DFR: Dihydroflavonol 4-reductase
F3′H: flavonoid 3′-hydroxylase
F3′5′H: flavonoid 3′5′-hydroxylase.

## References

[B1] Aida R. Yoshida K. Kondo T. Kishimoto S. Shibata M. (2000). Copigmentation gives bluer flowers on transgenic torenia plants with the antisense dihydroflavonol-4-reductase gene. Plant Sci. 160, 49–56. 10.1016/S0168-9452(00)00364-2 11164576

[B2] Davies K. M. (ed.). (2004). An introduction to plant pigments in biology and commerce, in *Plant Pigments and their Manipulation* (Oxford: Blackwell Publishing), 1–22.

[B3] Dellus V. Heller W. Sandermann H. Scalbert A. (1997). Dihydroflavonol 4-reductase activity in lignocellulosic tissues. Phytochemistry 45, 1415–1418. 10.1016/S0031-9422(97)00128-3

[B4] Feng H. Tian X. Liu Y. Li Y. Zhang X. Jones B. J.. (2013). Analysis of flavonoids and the flavonoid structural genes in brown fiber of upland cotton. PLoS ONE 8:e58820. 10.1371/journal.pone.0058820 23527031 PMC3602603

[B5] Forkmann G. Heller W. (1999). Biosynthesis of flavonoids, in *Polyketides and Other Secondary Metabolites Including Fatty Acids and Their Derivatives*, Vol. 1, ed Sankawa U. (Amsterdam: Elsevier), 713–748.

[B6] Fukui Y. Tanaka Y. Kusumi T. Iwashita T. Nomoto K. (2003). A rationale for the shift in colour towards blue in transgenic carnation flowers expressing the flavonoid 3′,5′-hydroxylase gene. Phytochemistry 63, 15–23. 10.1016/S0031-9422(02)00684-2 12657292

[B7] Gosch C. Nagesh K. M. Thill J. Miosic S. Plaschil S. Milosevic M.. (2014). Isolation of dihydroflavonol 4-reductase cDNA clones from Angelonia x angustifolia and heterologous expression as GST fusion protein in *Escherichia coli*. PLoS ONE 9:e107755. 10.1371/journal.pone.0107755 25238248 PMC4169556

[B8] Grotewold E. (2006). The genetics and biochemistry of floral pigments. Annu. Rev. Plant Biol. 57, 761–780. 10.1146/annurev.arplant.57.032905.105248 16669781

[B9] Helariutta Y. Elomaa P. Kotilainen M. Seppänen P. Teeri T. H. (1993). Cloning of cDNA coding for dihydroflavonol-4-reductase (DFR) and characterization of dfr expression in the corollas of Gerbera hybrida var. Regina (Compositae). Plant Mol. Biol. 22, 183–193. 10.1007/BF00014927 8507822

[B10] Holton T. A. (1996). Transgenic Plants Exhibiting Altered Flower Color and Methods for Producing Same. Patent No. PCT/AU96/00296.

[B11] Honda T. Saito N. (2002). Recent progress in the chemistry of polyacylated anthocyanins as flower color pigments. Heterocycles 56, 633–692. 10.3987/REV-01-SR(K)2

[B12] Hua C. Linling L. Shuiyuan C. Fuliang C. Feng X. Honghui Y.. (2013). Molecular cloning and characterization of three genes encoding dihydroflavonol-4-reductase from Ginkgo biloba in anthocyanin biosynthetic pathway. PLoS ONE 8:e72017. 10.1371/journal.pone.0072017 23991027 PMC3753345

[B13] Johnson E. T. Ryu S. Yi H. Shin B. Cheong H. Choi G. (2001). Alteration of a single amino acid changes the substrate specificity of dihydroflavonol 4−reductase. Plant J. 25, 325–333. 10.1046/j.1365-313x.2001.00962.x 11208024

[B14] Katsumoto Y. Fukuchi-Mizutani M. Fukui Y. Brugliera F. Holton T. A. Karan M.. (2007). Engineering of the rose flavonoid biosynthetic pathway successfully generated blue-hued flowers accumulating delphinidin. Plant Cell Physiol. 48, 1589–1600. 10.1093/pcp/pcm131 17925311

[B15] Meyer P. Heidmann I. Forkmann G. And Saedler H. (1987). A new petunia flower colour generated by transformation of a mutant with a maize gene. Nature 330, 677–678. 10.1038/330677a0 3683587

[B16] Petit P. Granier T. d'Estaintot B. L. Manigand C. Bathany K. Schmitter J. M.. (2007). Crystal structure of grape dihydroflavonol 4-reductase, a key enzyme in flavonoid biosynthesis. J. Mol. Biol. 368, 1345–1357. 10.1016/j.jmb.2007.02.088 17395203

[B17] Shimada N. Sasaki R. Sato S. Kaneko T. Tabata S. Aoki T.. (2005). A comprehensive analysis of six dihydroflavonol 4-reductases encoded by a gene cluster of the Lotus japonicus genome. J. Exp. Bot. 56, 2573–2585. 10.1093/jxb/eri251 16087700

[B18] Tanaka Y. Brugliera F. (2006b). Flower colour, in *Flowering and its Manipulation*, ed Ainsworth C. (Oxford: Blackwell), 201–239.

[B19] Tanaka Y. Fukui Y. Fukuchi-Mizutani M. Holton T. A. Higgins E. Kusumi T. (1995). Molecular cloning and characterization of *Rosa hybrida* dihydroflavonol 4-reductase gene. Plant Cell Physiol. 36, 1023–1031. 8528604 10.1093/oxfordjournals.pcp.a078844

[B20] Tanaka Y. (2006a). Flower colour and cytochromes P450. Phytochem. Rev. 5, 283–291. 10.1007/s11101-006-9003-724709279

[B21] Xie D. Y. Jackson L. A. Cooper J. D. Ferreira D. Paiva N. L. (2004). Molecular and biochemical analysis of two cDNA clones encoding dihydroflavonol-4-reductase from *Medicago truncatula*. Plant Physiol. 134, 979–994. 10.1104/pp.103.030221 14976232 PMC389921

[B22] Yoshida K. Mori M. Kondo T. (2009). Blue flower color development by anthocyanins: from chemical structure to cell physiology. Nat. Prod. Rep. 26, 884–915. 10.1039/b800165k 19554240

[B23] Zhu S. W. Gao P. Sun J. S. Wang H. H. Luo X. M. Jiao M. Y. (2006). Genetic transformation of green-colored cotton. In Vitro Cell. Dev. Biol. Plant 42, 439–444. 10.1079/IVP2006777

